# Using long-term datasets to assess the impacts of dietary exposure to neonicotinoids on farmland bird populations in England

**DOI:** 10.1371/journal.pone.0223093

**Published:** 2019-10-01

**Authors:** Rosie J. Lennon, Nick J. B. Isaac, Richard F. Shore, Will J. Peach, Jenny C. Dunn, M. Glória Pereira, Kathryn E. Arnold, David Garthwaite, Colin D. Brown

**Affiliations:** 1 Department of Environment and Geography, University of York, York, England, United Kingdom; 2 Centre for Ecology & Hydrology, Wallingford, Oxfordshire, England, United Kingdom; 3 Centre for Ecology & Hydrology, Lancaster Environment Centre, Lancaster, England, United Kingdom; 4 RSPB Centre for Conservation Science, Royal Society for the Protection of Birds, Sandy, Bedfordshire, England, United Kingdom; 5 School of Life Sciences, Joseph Banks Laboratories, University of Lincoln, Lincoln, England, United Kingdom; 6 Fera Science Ltd., National Agri-food Innovation Campus, Sand Hutton, York, England, United Kingdom; University of Bern, SWITZERLAND

## Abstract

Over the last 20 years, a new group of systemic insecticides–the neonicotinoids—has gained prominence in arable systems, and their application globally has risen year on year. Previous modelling studies using long-term data have suggested that neonicotinoid application has had a detrimental impact on bird populations, but these studies were either limited to a single species or neglected to analyse specific exposure pathways in conjunction with observed population trends. Using bird abundance data, neonicotinoid usage records and cropping data for England at a 5x5 km resolution, generalised linear mixed models were used to test for spatio-temporal associations between neonicotinoid use and changes in the populations of 22 farmland bird species between 1994 and 2014, and to determine whether any associations were explained by dietary preferences. We assigned farmland bird species to three categories of dietary exposure to neonicotinoids based on literature data for species diets and neonicotinoid residues present in dietary items. Significant estimates of neonicotinoid-related population change were obtained for 13 of the 22 species (9 positive effects, 4 negative effects). Model estimates for individual species were not collectively explained by dietary risk categories, so dietary exposure to neonicotinoids via ingestion of treated seeds and seedlings could not be confirmed as a causal factor in farmland bird declines. Although it is not possible to infer any generic effect of dietary exposure to neonicotinoids on farmland bird populations, our analysis identifies three species with significant negative estimates that may warrant further research (house sparrow *Passer domesticus*, skylark *Alauda arvensis* and red-legged partridge *Alectoris rufa*). We conclude that there was either no consistent effect of dietary exposure to neonicotinoids on farmland bird populations in England, or that any over-arching effect was not detectable using our study design. The potential for indirect effects of insecticide use on bird populations via reduced food availability was not considered here and should be a focus for future research.

## Introduction

Agricultural intensification is thought to be the largest threat to global avifauna [1]. Significant declines in farmland birds have been well documented over the past 30 years and have been attributed to many aspects of agricultural intensification, including habitat loss, seasonal shifts in cultivation practices and the increased use of agro-chemicals [2, 3]. A recent review of farmland bird declines in North America found that pesticide use was the most commonly reported driver of population declines in farmland birds (42% of all studies, 93% of which reported negative impacts), followed by habitat loss and alterations [2]. Similarly, insecticide application was found to be one of the higher ranking variables to explain farmland bird declines during agricultural intensification in the UK between 1962 and 1995 [3] and has been cited in multiple reports as one of the key agricultural practices that has contributed to avian population change [4–6].

**Fig 1:**
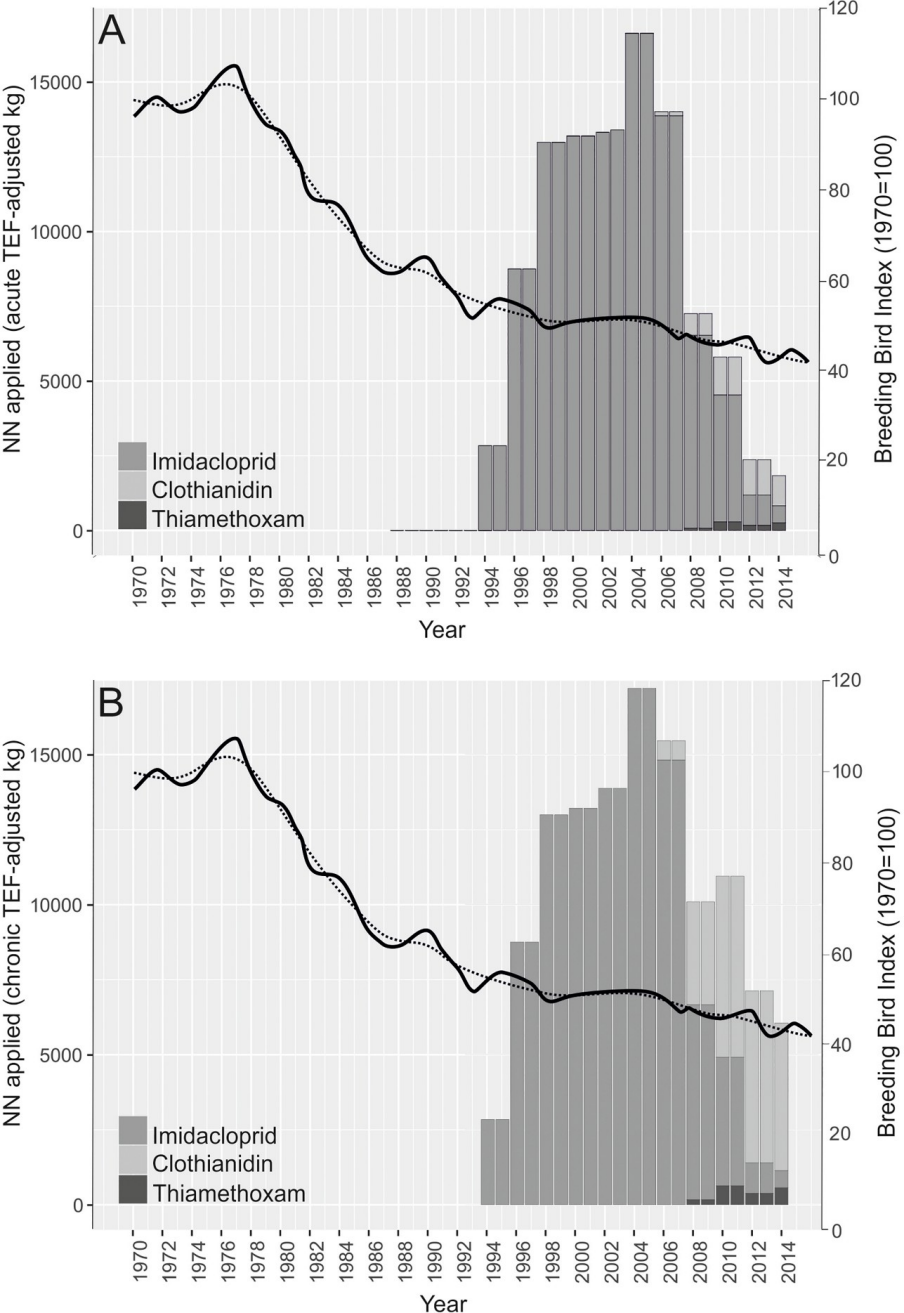
Change in NN application and change in farmland bird abundance for the UK between 1970 and 2014. Bars: Pesticide Usage Survey data for annual weight (kg) of NN applied, moderated by a toxicity equivalency factor (TEF) to account for differences in the acute (**Fig 1A**) or chronic (**Fig 1B**) toxicity of each NN compound to birds (see Methods for details) [9]. Lines: breeding bird index for farmland birds based on 19 farmland indicator species (solid: unsmoothed trend; dotted: smoothed trend), reproduced from the Defra report ‘Wild bird populations in the UK, 1970 to 2014: Annual statistical release’ (Fig 2) [12]. NN: neonicotinoid.

Over the last 20 years, the neonicotinoid (NN) group of systemic insecticides has gained prominence in arable systems, and their application globally has risen year on year [7]. Over 90% of NN applications in the UK (based on area treated) have been in the form of coated seed [8] with imidacloprid (IMI), clothianidin (CTD) and thiamethoxam (THX) the three most commonly used compounds [9]. In the UK there has been a significant shift in the main compound of use during the period of NN application. Prior to 2008, IMI was the main compound applied as seed treatment, but from 2008 onwards CTD took precedence. NN compounds also differ in their toxicity to birds [10]; in bobwhite quail *Colinus virginianus* IMI is over 13-times more toxic than CTD [11]. As a result, both acute and chronic toxicity to birds in the UK (theoretically) peaked in the mid-2000s (**Fig 1A** and **Fig 1B**, respectively), rather than mirroring the net weight of NN applied (**S1 Fig**). Patterns of NN usage corrected for either acute or chronic toxicity are identical through to mid-2000s, but there is a slower decline from that peak when correcting for chronic toxicity (Fig 1B) because the difference in toxicity between IMI and the other NNs is smaller for chronic exposure than for acute exposure.

UK farmland bird populations declined substantially between 1970 and 2013. Of the 19 farmland indicator species (those deemed dependent on farmland habitat), 12 experienced population declines of between 23 and 97% [13]. The steepest declines took place between the mid-1970s and the early-1990s (**Fig 1**) when the amount of farmland hedgerow had decreased significantly, a widespread switch to autumn sowing occurred, and the number of commercial pesticides in use (including DDT up until it was banned in 1986) rose from 137 to 344 as a result of agricultural intensification [14]. NNs were first used as agricultural plant protection products in Britain in 1994 [15] at a time when farmland bird declines appeared to slow. Nevertheless, there are growing concerns within the scientific community regarding the availability of NNs to birds and the potential for effects of NNs on avian physiology and behaviour [11, 16–21].

According to manufacturers’ instructions, NN-treated seeds should be efficiently incorporated at drilling to minimise exposure to non-target species [22]. However, recent research in Spain found a mean (± SE) of 43.4 ± 5.5 seeds per m^2^ on field headlands within the first two weeks following NN applications [16]; this suggests that the risk posed from availability and subsequent ingestion of seeds by birds may have been underestimated. Furthermore, NN residue has also been detected in crop seedlings, which are thought to take up approximately 1–15% of compound applied to seed coatings [23, 24], and wild plants at field boundaries [25]. Crop seedlings and vegetation at agricultural margins provide food for a number of farmland bird species, suggesting another potential pathway of exposure to NNs.

Thus far, only a handful of studies have investigated pathways of exposure to NNs for farmland birds, and the primary focus for granivorous birds has been on ingestion of NN-treated seeds. Prosser (2001) recorded a total of 18 species foraging on seed types that are regularly treated with NNs as part of agricultural practice [26] and Lopez-Antia *et al*. (2016) observed 30 species consuming NN-treated seeds in recently drilled fields [16]. Furthermore, NN residues have been detected in two wild passerine species [20, 27], and in the eggs, crops and livers of wild partridges [28, 29]. A detailed review conducted by the American Bird Conservancy calculated that as few as 3.9 and 1.3 imidacloprid-coated wheat seeds could produce lethal and sub-lethal (reproductive) effects, respectively, if ingested by a 15-g bird [11]. There is also potential for direct ingestion of NN-contaminated insects as many granivorous bird species will switch to an insectivorous diet during the breeding season; however, the relatively small concentrations of NNs on insects [30] means that ingestion of NN-treated seeds and seedlings is likely to be a much more significant source of exposure. Various aviary experiments have found that birds dosed with environmentally-relevant concentrations of NNs can suffer changes to the immune system, oxidative stress, impaired navigational ability and the accumulation of NN residues in the liver [18, 21, 31]. Thus not only is it possible for birds to be exposed to NNs, but the likely levels of exposure may be sufficient to produce sub-lethal effects and these may in turn affect survivorship, reproduction and consequently, populations.

Even though the literature identifies the potential for effects of NNs on farmland birds, there is a sparsity of evidence on whether bird populations have actually been impacted. In 2014, a Dutch study investigated the spatial correlation between surface water concentrations of NNs and insectivorous bird population trends, and reported that in areas where IMI concentrations in water were >20 ng/L, bird populations experienced average annual declines of 3.5% across 15 insectivorous species [32]. The study postulated that the observed trends were a result of depleted insect food resources, occurring as a result of NN-usage. However, despite the thorough statistical approach used for these analyses, the causative link between surface water concentrations and population level impacts remained hypothetical. A separate study evaluated effects of historic NN use on abundance of bobwhite quail in Texas by developing models structured by time period (pre- or post-NN use) and eco-region, including potential confounding variables such as temperature, land use and precipitation (32). NN use was found to be the variable that most commonly exhibited a negative association with quail abundance (62% of all post-NN use models), although a causative pathway by which NN use may have impacted quail populations was not defined. As yet, there are no long-term studies that investigate explicitly whether dietary exposure to NNs has been associated with population-scale effects on birds.

In the present study, we hypothesise that dietary exposure to NNs via ingestion of treated seed and/or crop material is associated with population declines of granivorous farmland birds. To gain adequate power to test this hypothesis, we construct a model with 21 years of pesticide usage and bird abundance data for England expressed at a 5x5 km resolution. This model is used to test: 1) whether spatio-temporal variation in NN use over a 21-year period is correlated with changes in the abundance of 22 individual farmland bird species; and 2) whether any correlations that exist are associated with potential dietary exposure to NNs based on known dietary preferences of the individual bird species. This is the first analysis of its kind to focus on farmland bird populations with regards to the long-term application of a specific pesticide group and a specific dietary route of exposure.

## Methods

**Fig 2:**
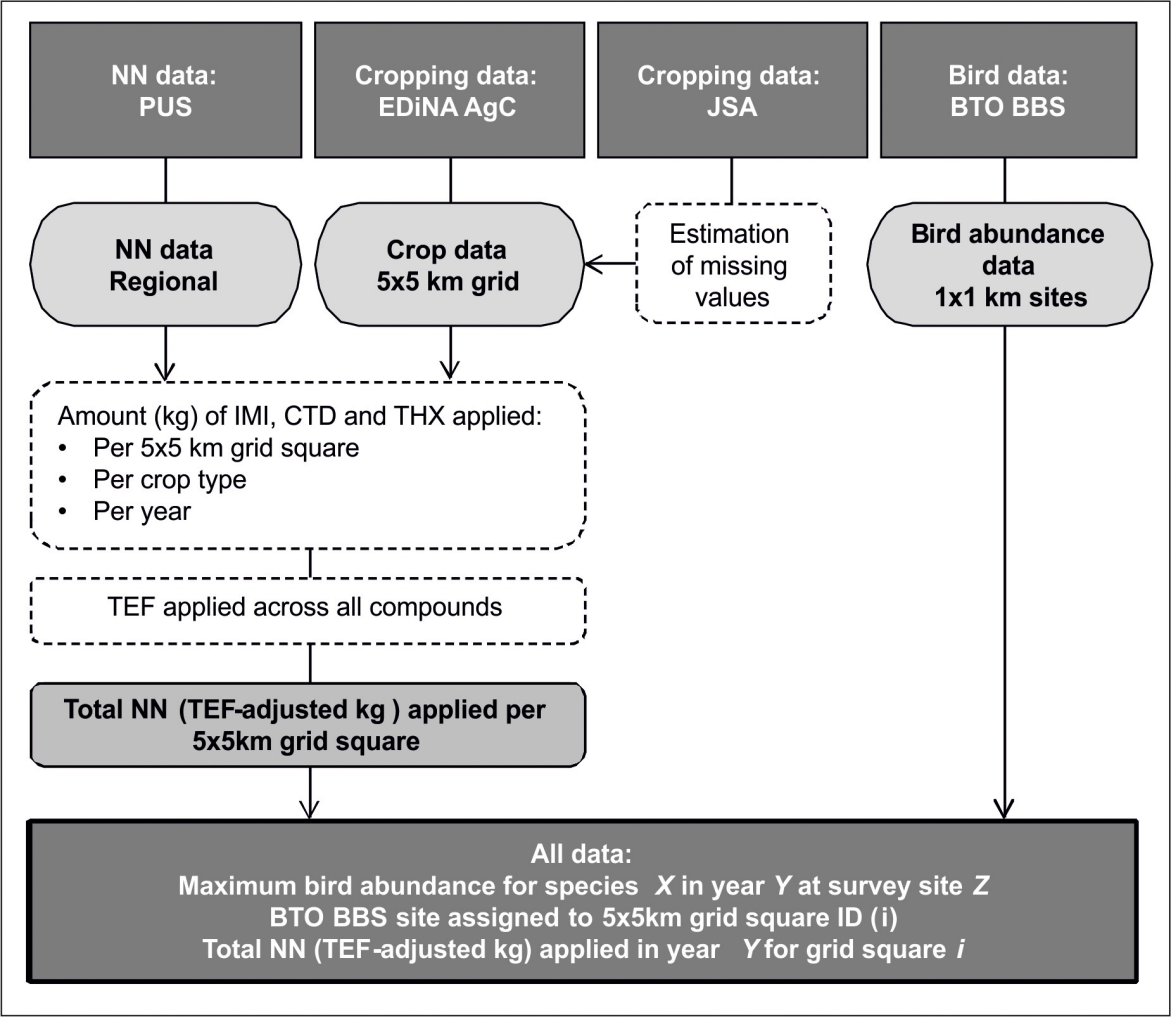
Overview of the manipulation process used to combine independent data sources to build the final model data frame. AgC: EDiNA agcensus; BBS: breeding bird survey; BTO: British Trust for Ornithology; CTD: clothianidin; IMI: imidacloprid; JSA: June Survey of Agriculture; NN: neonicotinoid; PUS: Pesticide Usage Survey; TEF: toxicity equivalency factor (used to adjust for the differences in toxicity of each compound to birds); THX: thiamethoxam.

Three datasets comprising bird abundance, NN usage and cropping data (each resolved to a 5x5km resolution) were used to build the model to test our hypotheses. These data were obtained from the British Trust for Ornithology (BTO) Breeding Bird Survey (BBS) [33], the pesticide usage surveys (PUS) [9] and the EDiNA agcensus (AgC) dataset [34], respectively. An overview of the data manipulation process used in producing the data frame for analysis is given in **Fig 2**.

### Calculating spatial NN application rates for England: 1994–2014

Pesticide usage data were only available at a regional level (approximately 20,000 km^2^). Here annual NN application at a 5x5 km scale was interpolated using spatial cropping data [35].

#### Cropping data

Cropping data were obtained for England from the EDiNA AgC resource at a 5x5 km scale. Data were obtained for all available years from 1994 to 2014, and for all crops identified by the PUS as receiving NN applications as a seed coating. Sufficient data were available for all major arable crop types except rye (*Secale sp*.; **Table 1**). A total of 9221 AgC 5x5km grid squares were available for England. Each grid square was assigned a ‘NUTS’ region based on level 1 of the Nomenclature of Territorial Units for Statistics (NUTS; 9 regions for England), and a ‘Defra’ region (5 regions for England) to match with the two types of region categories used in the PUS dataset (1994–2002: Defra regions; 2004–2014: NUTS regions; **S2 Fig**).

**Table 1:** Availability of EDINA agcensus data for each crop type in England.

Crop	Genus	Missing Years	Interpolation method for missing years*
Sugar beet	*Beta*	1998;1999;2001;2002;2006–2009; 2011–2014	Linear
Oilseed rape	*Brassica*	1998;1999;2001;2002;2006–2009; 2011–2014	Linear, Regional JSA
Wheat	*Triticum*	1998;1999;2001;2002;2006–2009; 2011–2014	Regional JSA
Winter Barley	*Hordeum*	1998;1999;2001;2002;2006–2009; 2011–2014	Regional JSA
Linseed	*Linum*	1998;1999;2001;2002;2006–2009;2011–2014	Regional JSA, National JSA
Oats	*Avena*	1998;1999;2001;2002;2006–2009; 2011–2014	Regional JSA
Rye	*Secale*	1998–2014	None: excluded from analysis

*No interpolation for 1998 due to non-availability of JSA and agcensus data across all crop types.

JSA: June Survey of Agriculture (Defra).

As there was a significant number of consecutive missing years for cropping data, regional data obtained from the June Survey of Agriculture (JSA) were used to estimate the areas of individual crops within each grid square for all missing years (national JSA data were also used for linseed [*Linum sp*.] where regional data were not available). Where JSA data were not available for a missing year, linear interpolations were used to estimate cropping areas per grid square (**Table 1**). Details of interpolation methods can be found in **S1 Supplementary Note**. Cropping data were not available from either AgC (at a 5 x 5 km resolution) or JSA (at a regional resolution) for any crop type in 1998; this year was therefore excluded from the analysis.

#### NN data

Regional NN usage data were obtained from the PUS provided by FERA Science Ltd [9]. These data provided the weight (kg) of NN applied as seed treatments by crop type, year, and region, with the survey year denoting the year of harvest (i.e., autumn sowings of winter crops in year *n*-1 and spring sowings of spring crops in year *n* would both be counted in the survey for year *n*). Data were available for all arable crops in England at a two-year resolution from 1994 to 2014. For odd years (those with no data) pesticide usage values for each region and each crop type were calculated by taking the mean of values for the preceding and following years. The sensitivity of the model to this approach was tested using an alternative assumption that NN use in a year without data was the same as in the preceding year when data were collected.

#### NN application rate per grid square

Total compound application per 5x5 km grid square was calculated using **Eqs 1–3**:

$$\left(\frac{x}{y}\right)\times 100=Z$$ (Eq 1)

$$\left(\frac{A}{100}\right)\times Z=B$$ (Eq 2)

∑B(allcroptypes)=C (Eq 3)

where *x* = total crop area in grid square (ha), *y* = total crop area in region (ha), *Z* = percentage of total crop in region that the grid square contains, *A* = total amount of compound applied in region per crop (kg), B = total compound application per crop type (kg per grid square), and C = total NN application per grid square (kg).

A toxicity equivalency factor (TEF) was applied to account for differences between compounds in either their acute or chronic toxicity to birds. The acute TEF was based on the oral acute toxicity (LD_50_) for bobwhite quail for each compound (152, 2000 and 2716 ng/kg body weight for IMI, CTD, and THX, respectively [11]). The TEF for IMI was set at 1, and the TEFs for CTD and THX were calculated as 152/2000 (0.08) and 152/2716 (0.06), respectively. The chronic TEF used critical intake values for a sensitive bird at the 5% tail of the acute sensitivity distribution published by Mineau and Palmer [11] based on lowest observed adverse effect levels (2820, 7380 and 12660 ng/kg body weight/day for IMI, CTD and THX respectively, giving TEF values of 1, 0.38 and 0.22, respectively. Acute TEFs were multiplied by the application rates for each compound per grid square per year, and the values for each compound were summed to give the total TEF-adjusted NN (kg) applied per grid square for use in the primary analysis. A repeat analysis was undertaken using the chronic TEF values to investigate the impact that this had on model results.

### Bird data

BTO BBS data were obtained for 22 farmland species for the period 1994 to 2014 (**S1 Table**). The BBS consists of two visits per year (April/May and May/June) to a series of 1x1 km^2^ survey sites where all species seen and heard are recorded across 10 transects within the survey square. Here, the maximum species count from either visit was extracted per site and per year as the measure of bird abundance. Both audible and visual records were included across all BBS distance categories, including fly overs. All birds on the farmland bird indicator list (19 species native to the UK [36]) were included, as well as red-legged partridge (*Alectoris rufa*), which is a non-native farmland specialist. Data for house sparrow (*Passer domesticus*) and chaffinch (*Fringilla coelebs*) were also included due to the availability of appropriate dietary data for these granivorous species.

Only BBS sites for which the level 1 habitat type was specified as farmland (code: ‘E’), for the grid reference location of the BBS site, in one or more surveys during the time series were included in the analyses. A block of 343 sites in the North-West of England for which level 1 habitat type was not recorded were also included. Each BBS survey location (the central point of the 1 km square in which the BBS was undertaken) was assigned to the 5x5km grid square in which it fell. The analysis was restricted to BBS squares within mainland England to match the available pesticide and cropping data. All BBS data for 2001 were excluded from the analysis due to anomalies caused by site access restrictions during an outbreak of foot-and-mouth disease. Total change in each species population growth for England between 1995 and 2016 (referred to as ‘BBS trends’) was also independently obtained for each species from existing BTO BBS data sources [37] (**S1 Table**).

#### Defining NN exposure category for each species

The majority of bird species have heterogeneous diets [38–41], so data on dietary preferences were used to generate an index of likelihood of exposure. **Table 2** presents data for NN residue in potential food items, and a resulting categorisation of food items into low-level and high-level residue categories. Treated seed and crop seedlings represent food items with ‘high’ NN residue, while exposed birds (as prey items), eggs laid by exposed birds and exposed wild plant species were categorised as food items with ‘low’ NN residue (<0.01% of highest concentration). Invertebrates were found to have negligible NN residue (see **S2 Supplementary Note** for details) and were added to the ‘low’-level residue category.

**Table 2:** Reported concentrations of NN residues in avian dietary components.

Dietary component	Data source	Residue of NN (ng/g)	Compound	Residue level
Crop seed	RSPB (*pers*. *com*)	555,600	CTD	High
Crop seedlings	RSPB (*pers*. *com*)	3,425	CTD	High
Exposed birds (<50g)	Lopez-Antia *et al*. (2015)	56	IMI	Low
Eggs (exposed bird)	Bro *et al*. (2016)	28	IMI	Low
Wild plants (at field margins)	Biotas *et al*.(2016)	0.51	CTD	Low
Invertebrates*	Chauzat *et al*. (2011)	0.3–11.1	IMI	Low

*Concentrations recorded in field-sampled honeybees (Apis mellifera) [30]; see **S2 Supplementary Note**).

CTD: clothianidin; IMI: imidacloprid; NN: neonicotinoids.

Mean proportions of plant families in species diets were extracted from a quantitative literature review of European farmland bird diets reported by Holland *et al*. for 16 species [38] (**Table A** in **S3 Supplementary Note**). Where available, data were extracted for plant families *Cruciferae* (crops only), *Poaceae* (cereals only) and *Amaranthaceae* (all), which cover the main crop types associated with NN application (wheat, barley, sugarbeet, oilseed rape, rye and oats). Data were extracted separately for breeding and non-breeding adult birds and for chicks. Where specific plant family data were not available, values were estimated from data for the total percentage of plant material in species diets at each life stage (**S3 Supplementary Note**). Due to the variety of dietary assessment methods used in the studies reviewed in Holland *et al*. [38], extracted proportion values across multiple plant species were summed for each bird species to provide a measure of high-level residue food items in the diet (i.e., NN-treatable crop seed and seedling) and to capture the potential exposure from multiple crop types (**Table 3**).

**Table 3:** Relative quantity of high-level residue food items in species diet and dietary exposure groups assigned to each species.

Bird species	Latin name	Plant families treated with NN that are present in species diet	Relative value* (based on summed proportions) of plant families in diet at each life stage			Exposure group
			*Adult BR*	*Adult NB*	*Chicks*	
Chaffinch	*Fringilla coelebs*	*Poaceae (O)*	44	25	n/a	Medium
Corn Bunting	*Miliaria calandra*	*Poaceae*	44	75	16	High
Goldfinch	*Carduelis carduelis*	None	0	0	n/a	Low
Greenfinch	*Carduelis chloris*	*Poaceae*	16	11	21	Medium
Grey Partridge	*Perdix perdix*	*Poaceae*	12	28	21	Medium
House Sparrow	*Passer domesticus*	*Poaceae (O)*	37	23	^24	Medium
Jackdaw^+^	*Corvus monedula*	(Cereal grain)	n/a	n/a	(11)	Medium
Kestrel^+^	*Falco tinnunculus*	None	(0)	(0)	(0)	Low
Lapwing^+^	*Vanellus vanellus*	None	(0)	(0)	(0)	Low
Linnet	*Carduelis cannabina*	*Cruciferae; Poaceae (O)*	0	0	71	High
Red-legged Partridge	*Alectoris rufa*	*Amaranthaceae; Poaceae (O)*	n/a	44	^29	Medium
Reed Bunting	*Emberiza schoeniclus*	*Amaranthaceae (O); Poaceae*	0	69	^0	High
Rook	*Corvus frugilegus*	*Poaceae*	38	58	34	High
Skylark	*Alauda arvensis*	*Amaranthaceae; (Poaceae*^+^*)*	^#^22	36	^2	Medium
Starling~	*Sturnus vulgaris*	(Grain)	(0)	(51)	(0)	Medium
Stock Dove	*Columbus oenas*	*Cruciferae; Poaceae*	61	22	5	High
Tree Sparrow	*Passer montanus*	*Amaranthaceae; Poaceae (O)*	22	36	^15	Medium
Turtle Dove	*Streptopelia turtur*	*Amaranthaceae (O); Cruciferae; Poaceae*	99	n/a	70	High
Whitethroat^+^	*Sylvia communis*	None	(0)	(0)	(0)	Low
Woodpigeon	*Columbus palumbus*	*Cruciferae; Poaceae (O)*	50	45	^47	High
Yellow Wagtail^+^	*Motacilla flava*	None	(0)	(0)	(0)	Low
Yellowhammer	*Emberiza citrinella*	*Poaceae*	92	32	4	High

*Extracted from Holland *et al*., 2006 [38], with the exception of

Values in brackets extracted from: (^+^) Birds of the Western Palearctic [39] and (~) Tait *et al*., 1973 [40].

Values estimated from Holland *et al*., 2006 [38] are indicated as follows: (^#^)Breeding value extrapolated from non-breeding value based on percentage of plant material in breeding vs. non-breeding season; (^) chick value extrapolated from available adult diet data based on percentage of plant material in breeding vs. non-breeding season (**S3 Supplementary Note**).

^+^Adult skylark are also known to feed on leaves of cereal plants (*Poaceae*) [41], but representative mean proportions are not shown here.

(O): Data includes percentage occurrence, as well as percentage items and percentage biomass.

AV: average; BR: breeding; NB: non-breeding.

Holland *et al*. [38] did not provide diet composition data for jackdaw (*Corvus monedula*), kestrel (*Falco tinnunculus*), starling (*Sturnus vulgaris*), lapwing (*Vanellus vanellus*), yellow wagtail (*Motacilla flava*) or whitethroat (*Sylvia communis*). For these species, dietary data were extracted from relevant volumes of Birds of the Western Palearctic [39]. Lapwing, yellow wagtail and whitethroat are insectivorous species, and kestrel a predatory species, so do not consume either crop seed or seedlings and were therefore assigned values of zero for these food items. Data extracted for adult jackdaw, nestling jackdaw and nestling starling were preferentially taken from studies with the largest available sample size, comparable sample type, sampling location within the UK and annual (rather than seasonal) data [39] (**S3 Supplementary Note**). Data for adult starling were extracted from Tait *et al*., 1973 [40] (**S3 Supplementary Note**).

Species were broadly assigned to one of three dietary exposure categories (high, medium and low) based on the relative proportions of high-level residue food items in the diet. ‘High’ potential for exposure was assigned where high-level residue food items comprised >50% of the diet at any life stage (i.e., chick, breeding adult, non-breeding adult), ‘medium’ if diet comprised between 1 and 49% high-residue food items, and ‘low’ if those items were not present in the diet across any life stage. Comparable dietary data (e.g., summed proportion values of individual plant families in the diet) were not available for jackdaw and starling; however data obtained from sources outside of Holland *et al*. confirmed that crop seed is present in the diets of both species [39, 40] and therefore both were conservatively assigned to the medium exposure group.

### Statistical modelling

A total of 3774 grid squares were used in the analysis, containing 5729 BBS sites (413 BBS sites were excluded from the analysis due to lack of cropping data and 6377 grid squares were excluded due to lack of BBS data). All models were run in R using the ‘glmmTMB’ function in the ‘glmmTMB’ package [42]. A separate model was fitted for each species, then the parameter estimates from each species model were compared to test our hypotheses.

#### Species specific model: NN application & species population growth

Individual generalised log-linear mixed models (adapted from Freeman and Newson 2008) were used to estimate the effect of NN application on population growth for each of the 22 species (**Eq 4**):

$$\ln\left(\mu_{g,t,s,r}\right)=\beta_{0}+\beta_{1}\sum_{j=1}^{t-1}P_{g,r}+\beta_{2}\sum_{j=1}^{t-1}{R_{j}}_{}+x_{g}+y_{s}+z_{r}$$ (Eq 4)

where the response variable *μ* is the count of birds in a given grid square *g* (at 5x5 km resolution), in year *t*, at BBS site *s* and within region *r*. The expected value of *μ*_*g*,*t*,*s*,*r*_ was modelled as a function of NN application (*P*; TEF-adjusted kg) and the ‘background’ species population growth (*β*_2_) in the absence of NNs as fixed effects. Grid square number (*x*), BBS site (*y*) and region (*z*) were modelled as normally-distributed random effects with zero mean. Issues related to density dependence were circumvented by using raw abundance data as the response variable to calculate population growth [43].

In detail, *β*_0_ represents the estimate of the log abundance for the relevant bird species in 1994 (the baseline year: *P* = 0), for the average grid cell, region and survey site (with distribution errors and log link). *R* was entered as a binary matrix, the columns of which indicate the time period across which species population growth is calculated, where *j* is an index of year. *β*_2_ therefore represents a vector of parameters, one for each year from 1995 to 2014, each of which is an estimate of the population growth rate for that year (i.e., the ‘background’ population growth rate); for example, the estimated log abundance for *μ* for 1996, at an ‘average’ site is given by *β*_0_+*β*_2(1995)_+*β*_2(1996)_. The variable *P* denotes the pesticide, measured as ‘cumulative’ NN (TEF-adjusted kg) from the baseline year (1994) up to and including the year of observation, indexed by *j*; note that ‘cumulative’ in this instance refers to the pesticide term within the model that is used to track year-on-year change in NN use, and does not imply multi-year accumulation of pesticide in the environment. Parameter *β*_1_ introduces the effect of NN application on the population growth (*β*_2_), in a similar way to the model used in Baker *et al*. [44]. Entering NN application (*P*) as a cumulative value allows *β*_1_ to be interpreted as the change in population growth rate per unit application of NN (adjusted for toxicity of each NN compound to birds). Simply put, the model tests the relationship between the change in bird abundance between years *t*−1 and *t* (*β*_2_) and the NN application in to crops harvested in year *t*−1 (*β*_1_), with the estimate represented as a decimal fraction. Therefore under the study hypothesis a negative impact of NN application on species of farmland birds would be indicated by negative estimates for NN-related population growth (*β*_1_) for species in the high exposure category.

NN applications to spring crops (particularly sugar beet) predominated in terms of total mass applied during the first half of the study period (1994–2004), whereas NN applications in the second half of the study period (2005–2014) were greatest for winter oilseed rape and winter cereals. As such, the possible demographic mechanisms through which NN exposure would affect our modelling of BBS counts include both reduced productivity, and overwinter survival or subsequent recruitment into breeding populations.

#### Model fit

All species models were initially run using a Poisson distribution and tested for over-dispersion (ratio of sum of squares residuals: residual degrees of freedom > 1.5; ‘overdisp’ function [45]) and zero-inflation (root mean squared error comparison, log-likelihood tests and the ‘testzeroinflation’ function in DHARMAa [46]). Residual QQ-plots were visually inspected for each species model to check uniformity, and simulated residuals were plotted (‘simulateResiduals’ function in DHARMAa) to check model fit.

All species except kestrel and woodpigeon were modelled using a quasi-Poisson distribution to account for over-dispersion in the count data, although data for lapwing and starling remained over-dispersed despite this adjustment (over-dispersion ratio = 1.68 and 1.90, respectively). Kestrel was modelled using a Poisson distribution and woodpigeon a negative binomial distribution. The fitted residuals were sigmoidal for all species models with non-uniform residual tails. The residuals for the grey partridge model were the only exception in that the residuals significantly deviated from the fitted trend for over 60% of the predicted values. It was not possible to use scaling to address these issues for this species.

#### Multispecies models: Dietary exposure

*β*_1_ estimates and their standard errors were extracted from each species-specific model. The difference in *β*_1_ estimates between dietary exposure groups (high, medium, low) were analysed using Kruskal–Wallis one-way analysis of variance (‘kruskall.test’, [47]). In order to account for differences in dietary preferences at each individual life stage, weighted linear regressions were used to model *β*_1_ as a function of the proportion of high-level residue food items for adult diet during the breeding season, adult diet outside of the breeding season, and chick diet for each species. A weighted linear regression was also used to assess whether there was any association between NN-related population change and overall population trends in England (BBS 1995–2016) across all species. Estimate values for *β*_1_ were weighted by their corresponding standard errors. Linear regressions were run in R using the ‘lm’ function [47].

## Results

**Fig 3:**
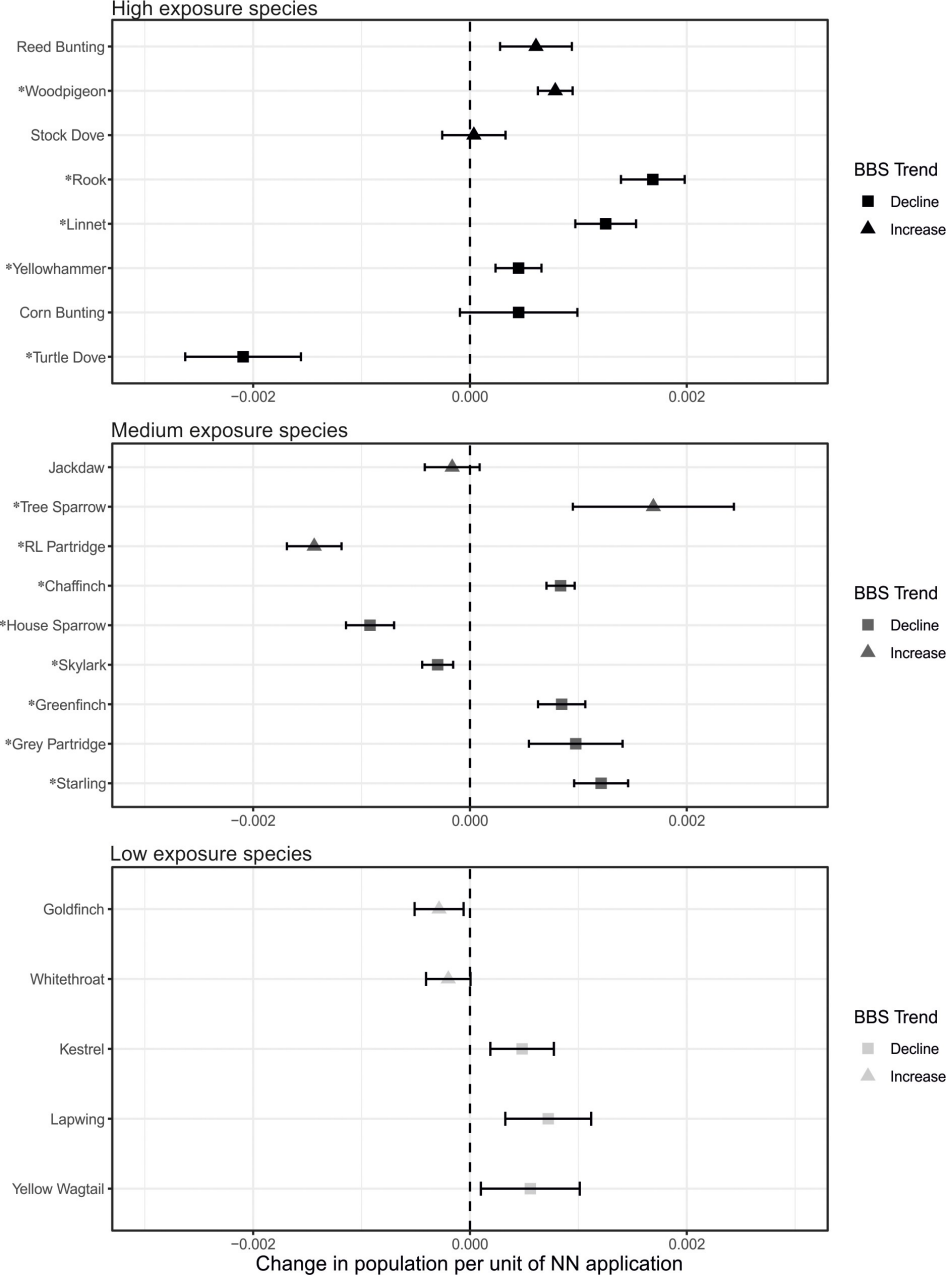
Model estimates plus standard error bars for change in species population per unit (TEF-adjusted kg) of NN applied for each species included in the analysis. Species are split by dietary exposure group and are ordered in each plot by rate of overall population change (‘BBS Trend’) according to BTO BBS data for England between 1995 and 2016 [37] with the largest population increase at the top and the largest decline at the bottom of each plot (see **S1 Table** for values). Species marked with (*) indicate significant (p<0.05) estimates of change in population per unit NN applied. BBS: breeding bird survey; BTO: British Trust for Ornithology; NN: neonicotinoid; TEF: toxicity equivalency factor.

Individual model estimates for the change in species population growth per unit (TEF-adjusted kg) of NN applied (*β*_1_—represented as a decimal fraction and referred to hereafter as ‘NN-related population change’) were obtained for all 22 study species (**S1 Table;** refer here for all Latin names hereafter), calculated across all years and all available grid squares. Estimates of NN-related population change (*β*_1_) ranged between -0.2 and +0.2%, and were significant for 13 out of the 22 species (p < 0.05) (**Fig 3** and **S1 Table**). There were significant positive estimates for nine species (chaffinch, greenfinch, grey partridge, linnet, rook, starling, tree sparrow, woodpigeon, yellowhammer), and significant negative estimates for four species (house sparrow, red-legged partridge, skylark, turtle dove). Standard errors in the estimate of *β*_1_ were largest for those species with fewest observations per survey event, in particular corn bunting, turtle dove and tree sparrow. BBS population trends for England (1995–2016) and NN-related population change were directionally matched for only seven of the 22 species (three species with negative BBS trends and *β*_1_ estimates, and four species with positive BBS trends and *β*_1_ estimates) (**Fig 3**). The root mean squared error was >10 for the majority of flocking species (jackdaw, rook, starling, woodpigeon) and <10 for those that are usually recorded in small numbers during the summer months (**S1 Table**). Overall, BBS site was the largest source of variance in the model for 18 of the 22 species, followed by grid square and region. For grey partridge, red-legged partridge, wood pigeon and yellow wagtail, grid square ID was the largest source of variance. Model outputs were almost identical when an alternative approach was used to estimate NN use in years without data (i.e., when data were repeated from the preceding year, rather than calculating the mean of the preceding and following years; **S2 Table**). Similarly, model outputs were almost identical when chronic TEFs were used to account for differences in toxicity between compounds rather than acute TEFs; there was a roughly equal split between species where the results shift towards a slightly more positive model estimate for NN effects on population size and those where the reverse was true (**S2 Table**); the estimate of negative impacts for the skylark changed to being non-significant in the analysis based on chronic TEFs, and the positive estimate for the reed bunting became significant.

Where NNs were applied, the median estimated value of application per grid square was 0.28 kg, with a maximum application of 69.98 kg (with TEF applied). The East region had the largest mean and total NN application over the entire study period, whilst the North West had the smallest (**S3 Table**).

### Dietary exposure & population change as a result of NN application

**Fig 4:**
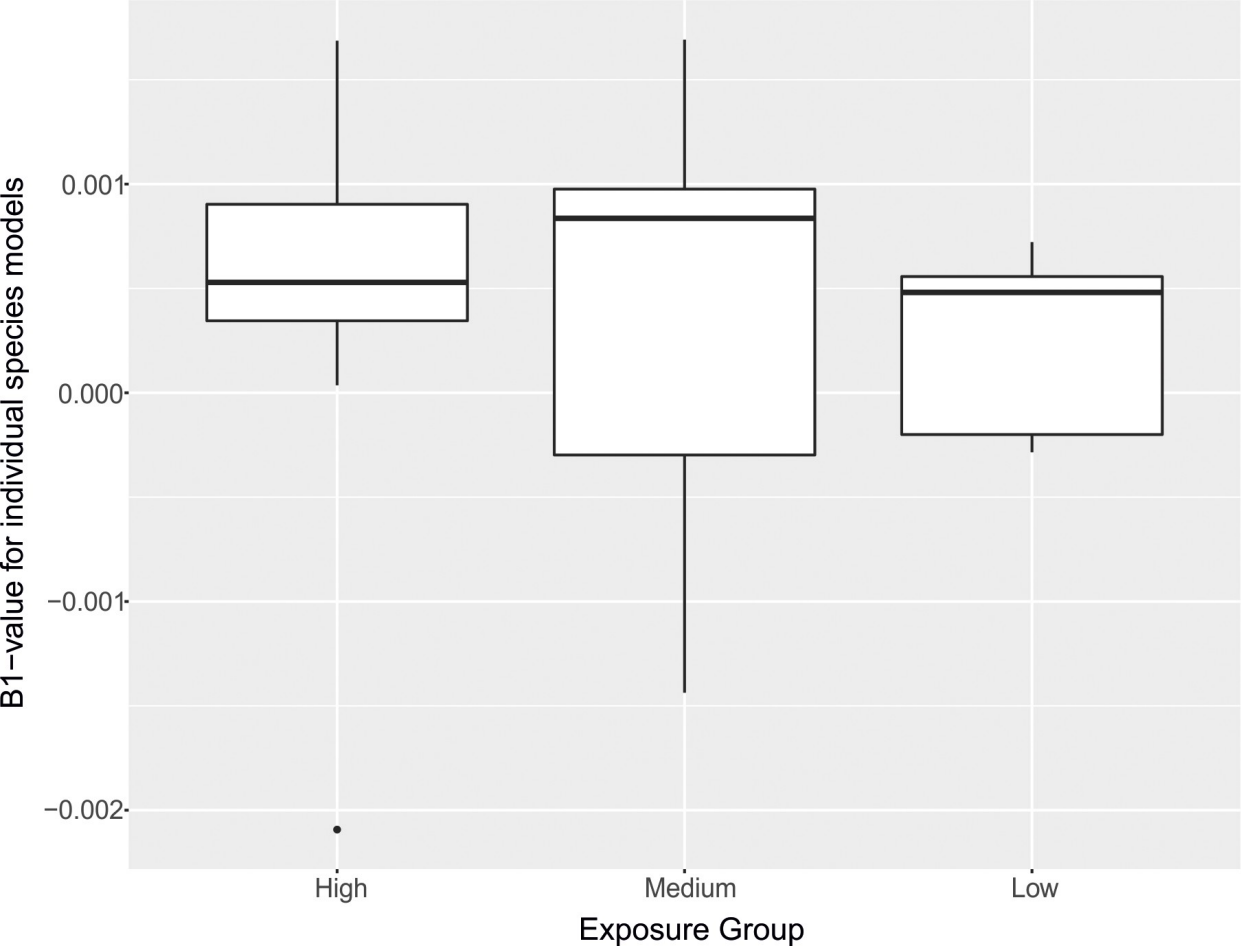
**Distribution of β_1_ values (change in species population growth per unit [TEF-adjusted kg] of NN applied) obtained for each species across dietary exposure groups**. The mean is represented by the black lines through the centre of each bar, the upper and lower quartiles are contained within the box and the range is represented by the whiskers. The estimate for turtle dove (*Streptopelia turtur*) is displayed as an outlier (represented by the single point) for the high exposure group. TEF: toxicity equivalence factor.

NN-related population change did not differ significantly between dietary exposure groups (Kruskall-Wallis chi-squared = 0.55, 2 d.f., p = 0.75; **Fig 4**). Furthermore, estimates of NN-related population change were not correlated with the relative values of high-residue food items in the diet of breeding adults, non-breeding adults or chicks extracted from Holland *et al*. (breeding adults: adjusted R^2^ = -0.029, F_1,17_ = 0.47, p = 0.49; non-breeding adults: adjusted R^2^ = -0.053, F_1,17_ = 0.08, p = 0.77; chicks: adjusted R^2^ = -0.021, F_1,17_ = 1.38, p = 0.25). There was also no correlation between NN-related population change and BBS trends (overall change in species population in England between 1995 and 2016) across all species in the study (adjusted R^2^ = -0.03, F_1,20_ = 0.27, p = 0.60).

## Discussion

Overall, our findings provide no consistent evidence for impacts of dietary exposure to NN insecticides on the abundance of farmland birds in England. Individual estimates of NN-related population change for each species varied considerably within the range of model outputs, but were noticeably smaller than annual ‘background’ changes in population for each species. Across all species, significant population change associated with spatial and temporal variation in NN application were mostly positive (9 out of 22), with a smaller number of negative relationships (4 out of 22). Under the study hypothesis, species in the high and the medium exposure groups were expected to have a higher proportion of significant negative estimates for NN-related population change compared to species in the low exposure group. Species in the low exposure group did not have any significant estimates of NN-related population change, which lends some support to the hypothesis. However, only one species in the high exposure group and three in the medium exposure group exhibited significant negative estimates. Moreover, nine species from these groups had significant positive estimates.

### Individual species

Of the nine species that had significant positive estimates for NN-related population change, four were in the high exposure category (linnet, rook, wood pigeon, yellowhammer), whilst the remaining five belonged to the medium exposure group (chaffinch, greenfinch, grey partridge, starling, tree sparrow). Seven of these nine species experienced population declines in England between 1995 and 2016. The most notable of these were grey partridge, linnet, and rook, (estimated declines of -58, -19, and -13%, respectively [37]). The remaining two species experienced population increases (tree sparrow: +64% and woodpigeon: +36%). However, estimates for rook, starling and woodpigeon had associated root mean squared error values (the number of birds per grid square by which the model estimate could vary) between 21 and 28, compared to <10 for the majority of other species. Rook, starling and woodpigeon in particular tend to form flocks, which may have added to the noise associated with the data for these species, especially with regard to ‘fly over’ records that may have recorded long-distance traveling flocks rather than local populations in each grid square. Furthermore, the model for starling was over- dispersed and the model fit for grey partridge was poor compared to all other species models. Thus, only five of the nine models reporting positive estimates for *β*_1_ were without confounding issues.

Positive estimates of NN-related population change for these nine species do not support the study hypothesis of adverse population change in response to dietary exposure to NNs. Currently, there is little evidence of a positive effect of NNs on birds in existing literature, and there is no known mechanism by which this could occur. One plausible explanation for these observed trends is that the overall availability of seeds/grain as a food resource within arable landscapes may have been strongly correlated with NN application, particularly at the height of NN use when a large proportion of crop types and large cropping areas were treated with NNs [9], resulting in greater granivorous species abundance at these sites. This theory is one that the present study cannot substantiate, but may be important to note as a potential paradox in NN exposure-population modelling of this type.

The four species that had significant negative estimates for NN-related population change were house sparrow, skylark, red-legged partridge and turtle dove. Of these, one was placed in the high exposure group (turtle dove), three belonged to the medium-exposure group (house sparrow, red-legged partridge, skylark), and all except red-legged partridge experienced overall population declines in England between 1995 and 2016. It is possible that the negative estimates for these species may be indicative of a true negative relationship between NN application and population change; indeed, a recent study reported widespread exposure of house sparrow to NNs in the field [27], but the implications of this exposure for fitness and/or survival were not assessed. However, other ecological factors may have also been important drivers. For instance, turtle dove populations are estimated to have undergone the greatest population decline of any species included in the study (-94%); however, turtle doves are migratory and unlikely to be exposed to NNs during the autumn sowing period as most individuals depart the UK in September at latest [48], and peak NN application occurs during late September and October [9]). Thus far, turtle dove population declines in the UK have primarily been attributed to the loss of weed seeds due to herbicide usage, resulting in an increased reliance on cultivated species such as cereals [49, 50].

The model output for red-legged partridge is also of note. Partridges (as well as other game birds) are one of the most commonly studied species in relation to NNs and exposure of various partridge species to NN-dressed seeds has been recorded [28, 29, 51, 52]. Sub-lethal impacts on red-legged partridge have been found when individuals have been given environmentally-relevant doses of IMI [53] while a long-term study found a significant negative impact of NNs on the population of the Northern bobwhite quail—another ground-dwelling galliform [54]. Our finding of a negative impact on red-legged partridge populations arising from NN use is therefore plausible when considered alongside previous research. However, there was a small population increase over the study period (+3% between 1995 and 2016 [37]) that indicates that other factors were likely to have been more important in determining population dynamics. Furthermore, this species is highly managed as part of the shooting industry, which may obscure natural changes in population numbers.

Collectively, our model outputs did not provide any consistent evidence that dietary exposure to NNs has had a negative impact on farmland bird populations in England at a 5x5 km spatial scale. We found that there were both significant positive and negative changes to individual species population growth where NNs were applied. It is unlikely that positive NN-related changes were directly related to NN use as there is no apparent mechanism by which NN ingestion is likely to be beneficial to birds (either individually or at a population scale). However, there is a substantial body of literature that provides evidence of NN-exposure to wild birds, and that NN ingestion results in adverse effects on avian physiology and behaviour [55]. We therefore cannot rule out the possibility that NN use had a negative effect on some species populations (particularly house sparrow, red-legged partridge and skylark) where negative changes were observed in areas where NNs were applied.

### Direct ingestion of NNs as an exposure pathway

Our exposure categories did not predict the magnitude of estimates for NN-related population change across the set of species included in the study; results here suggest that dietary exposure to NNs via treated seed and seedlings is unlikely to be associated with changes to farmland bird populations across England. Estimates of NN-related population change were both positive and negative within high and medium dietary risk groups and relative values of high-residue food items in the diet of adults and chicks did not explain population changes in the context of NN application. In addition, model estimates for four species in the high and medium risk groups were not significant (high risk group: corn bunting, stock dove and reed bunting; medium risk group: jackdaw), despite a large proportion of their diets consisting of high-residue food items. Corn bunting in particular has been cited in the literature as being a candidate species for studying the effect of NNs on small song birds due to the frequency with which it has been observed foraging in fields of treated seed [16], but this does not tally with our findings. The distribution of significant estimates between high and medium exposure categories suggests that NN-treated seed and seedling ingestion is not a strong driver of population change at this spatial scale (e.g., effects of NNs may be highly localised), and that NNs are uninfluential compared to other population drivers for the species included, such as food availably and habitat provision.

### Modelling approach

This analysis was undertaken with 19 years of pesticide usage and bird abundance data across 94,350 km^2^ (72%) of England. A key advantage in using these data is that the spatial and temporal variation in NN usage during the study period maximised the statistical power needed to test our hypotheses. Furthermore, our model verification process followed ‘best practice’ guidelines for fitting generalised linear mixed models [56]. Well-fitted models were difficult to achieve as is typical for many ecological studies using ‘real-world’ data collected from complex ecosystems. Nevertheless, the approach used is arguably one of the most powerful available to test our hypotheses.

In common with previous studies [32, 35], the spatial matching of NN usage data to records of non-target species required some interpolation of usage data. The model was shown not to be sensitive to the approach used to estimate NN usage in alternate years when pesticide usage data were not collected (**S2 Table**), but the interpolation step still introduces uncertainty into the analysis. The model structure also assumes that bird populations at each BBS site will only be affected by NN applications within the encompassing 5 x 5 km^2^ grid square. The hypotheses tested in this study related specifically to the ingestion of treated-crop material, whereas there are multiple exposure pathways that wild birds may be subject to. The decision to quantify NN in our model using weight of seed treatment applied means that exposure pathways associated with the much smaller usage of NNs as spray applications (~11% of applications in the UK during the study period [9]), such as direct overspray of birds or insects, were excluded from this study. However, these alternative pathways are expected to result in comparatively lower exposure than direct ingestion of treated seed or seedlings (**Table 2** and **S2 Supplementary Note**). Many granivorous birds switch to and/or feed their young an insectivorous diet during the breeding season [38] meaning there is also a potential impact on breeding success from reduced food availability [32]. This potential indirect impact from insecticide use was explicitly not considered within the current study and results should be interpreted in this context. The potential for indirect effects via reduced food availability would be a priority for future investigation and would require different measurements of NNs in the environment (e.g., residue in non-crop material or the impact of NNs on non-target invertebrate species). Finally, the analysis did not consider any particularly sensitive timings for NN application. As such, sub-lethal effects during the reproductive period were not specifically targeted, but were rather considered alongside the multiple sub-lethal endpoints proposed to result from neonicotinoid exposure in wild birds [19, 21, 31, 53] and which may affect both survival and productivity.

The overall number of species used in this study is both an advantage and a disadvantage. Modelling multiple species within one system allows for dietary exposure routes to be assessed through cross-species comparisons and is useful for pinpointing specific species from a large number of those potentially affected, which warrant further research attention. It also gives a full picture across a range of species with different physiologies, and different patterns of habitat use. The risk associated with modelling just one species is that, if a significant effect is found, it cannot be placed into context with either similar or dissimilar species, and that a finding for one species may be extrapolated to all species within that taxa. Conversely, the disadvantage of modelling multiple species is that the ‘one size fits all’ approach to the model structure may not be suitable across the board and may therefore contribute to poor model fit. Specifically tailored variables for each species may produce higher quality outputs (such as the approach used in Ertl *et al*., 2018), but at the cost of considerably narrowing the study spectrum.

### Conclusions

Here we found no evidence to suggest that dietary exposure to NNs via ingestion of treated seed and/or crop material has been associated with population declines of farmland birds in England over the period 1994 to 2014. We conclude that overall, there has either been no consistent effect of NN application on farmland bird populations, or any over-arching effect has been so small that it was not detectable. The potential for indirect effects of insecticide use on bird populations via reduced food availability was not considered within our study design and should be a focus for future research. This study highlights some of the issues in isolating specific causal factors for population dynamics from the ‘noise’ of other agricultural processes and underlying species population trends; this is particularly challenging when attempting to analyse a specific toxicant exposure route with regards to population-scale outcomes. Although it is not possible to infer any direct role of NNs on farmland birds collectively from these analyses, our results identify house sparrow, red-legged partridge and skylark as species that may warrant further research attention.

## Data sources

### Agcensus cropping data

The grid square agricultural census data, as converted by EDiNA at the University of Edinburgh and available through their AgCensus service (http://agcensus.edina.ac.uk), are derived from data obtained for recognised geographies from the Department of Environment, Food and Rural Affairs (DEFRA), the Welsh Assembly Government, and the Scottish Government (formerly SEERAD), and are covered by Crown Copyright.

### British Trust for Ornithology Breeding Bird Survey data

The Breeding Bird Survey (https://www.bto.org/volunteer-surveys/bbs/bbs-publications/bbs-reports) is run by the British Trust for Ornithology (BTO) and is jointly funded by the BTO, the Joint Nature Conservation Committee (JNCC) (on behalf of the statutory nature conservation bodies: Department of Agriculture, Environment and Rural Affairs—Northern Ireland, Natural England, Natural Resources Wales and Scottish Natural Heritage), and the Royal Society for the Protection of Birds (RSPB).

### Pesticide usage survey data

Fera Science Ltd is commissioned to conduct agricultural, horticultural and amenity pesticide usage surveys by the Chemicals Regulation Division (CRD) of the Health and Safety Executive. The surveys are funded from the pesticides charge on turnover, and the costs are paid to Fera Science Ltd by CRD. The Pesticide Usage Survey Teams of Fera Science Ltd, a joint venture between Capita PLC and the Department for Environment, Food & Rural Affairs (Defra), Science & Advice for Scottish Agriculture (SASA), a division of the Scottish Government’s Agriculture, Food and Rural Communities Directorate and the Agri-Food & Biosciences Institute (AFBI), a Non-Departmental Public Body of the Department of Agriculture and Rural Development, Northern Ireland (DARD) conduct a series of UK surveys of pesticide usage in the major sectors of agriculture and horticulture. Reports from these surveys are published on Fera’s website (https://secure.fera.defra.gov.uk/pusstats/surveys/index.cfm).

## Supporting information

S1 Fig Pesticide Usage Survey data for annual weight (kg) of NN applied in the UK between 1994 and 2014 (without toxicity equivalency factor applied) [9]. Bars are shaded according to amount of each NN compound annually applied. CTD: clothianidin; IMI: imidacloprid; THX: thiamethoxam; NN: neonicotinoid.
(PDF)

S2 Fig Pesticide usage survey regions. **(A)** Eight ‘NUTS regions’ (NUTS level 1) used in the pesticide usage survey from 2004 to 2014 (C: North East; D: North West; E: Yorkshire & Humber; F: East Midlands; G: West Midlands; H: Eastern; I&J: London & South East; K: South West). **(B)** Five ‘Defra regions’ (originally MAFF [Ministry of Agriculture, Fisheries & Food] regions) used in the pesticide usage survey from 1994 to 2002 (1: Northern; 2: Midlands & Western; 3: Eastern; 4: South East; 5: South West).
(PDF)

S1 Supplementary Note Interpolation method and validation. (PDF)

S2 Supplementary Note Concentrations of neonicotinoid in invertebrates. (PDF)

S3 Supplementary Note Data extraction protocol to inform dietary risk categories. (PDF)

S1 Table Summary of species diet (related to high-residue food items), species traits, model input and model output for each of the 22 species included in the analysis. (PDF)

S2 Table Alternative model outputs for each of the 22 species included in the study. **A) 'Stepped' interpolation for those years where pesticide surveys did not take place (all odd years).** Neonicotinoid usage was assumed to be the same in years where no data were available, as the preceding year (rather than being estimated by a linear interpolation). These data are presented as a means to test the sensitivity of the model to interpolation approaches used. **B) Chronic toxicity values used to calculate the toxicity equivalency factor (TEF). Chronic LOAEL values (at the 5% tail of acute sensitivity distribution for avian species) were used (rather than acute LD50 values for bobwhite quail *Colinus virginianus*) to calculate the TEF for the three compounds included in the study.** Calculations were based on information provided in Table 3.2 of Mineau & Palmer (2013). These data are presented as a means to test the sensitivity of the model to differences between acute and chronic TEFs.
(PDF)

S3 Table Estimated total application of NN (weight and TEF-adjusted weight) in each region for the entire study period (1994–2014). (PDF)
