# Supplementary material for: Using long-term datasets to assess the impacts of dietary exposure to neonicotinoids on farmland bird populations in England
Source: PLoS One. 2019 Oct 1;14(10):e0223093. doi: 10.1371/journal.pone.0223093 (PMC6772096; doi:10.1371/journal.pone.0223093)
Supplement: S1 Fig — Bars are shaded according to amount of each NN compound annually applied. CTD: clothianidin; IMI: imidacloprid; THX: thiamethoxam; NN: neonicotinoid. (PDF) [file pone.0223093.s001.pdf]

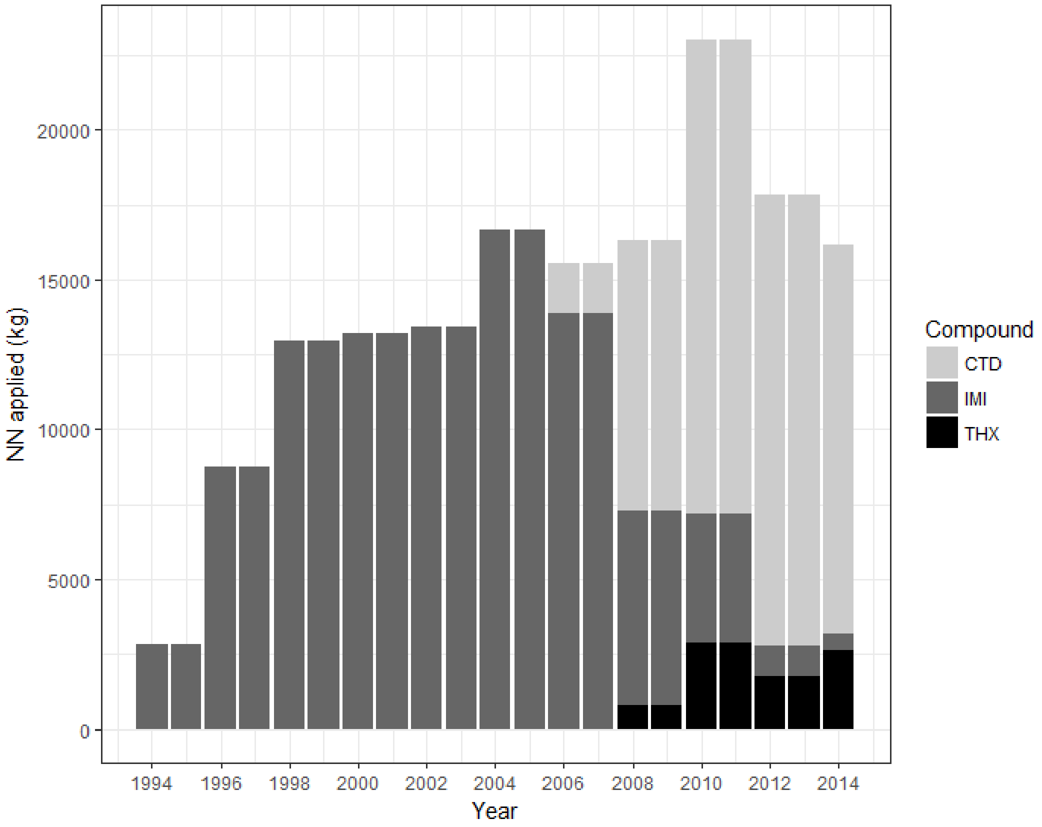

S1 Fig. Pesticide Usage Survey data for annual weight (kg) of NN applied in the UK between 1994 and 2014 (without toxicity equivalency factor applied) (1).

Bars are shaded according to amount of each NN compound annually applied. CTD: clothianidin; IMI: imidacloprid; THX: thiamethoxam; NN: neonicotinoid.

Reference

1. Garthwaite D, Hudson S, Barker I, Parrish GP, Smith L, Pietravalle S. Pesticide Usage Survey Report 255 – Grassland & Fodder Crops in Great Britain 2013. Food & Environment Research Agency (Fera Science Ltd.), UK; 2013.
