# Supplementary material for: Using long-term datasets to assess the impacts of dietary exposure to neonicotinoids on farmland bird populations in England
Source: PLoS One. 2019 Oct 1;14(10):e0223093. doi: 10.1371/journal.pone.0223093 (PMC6772096; doi:10.1371/journal.pone.0223093)
Supplement: S2 Fig — (A) Eight ‘NUTS regions’ (NUTS level 1) used in the pesticide usage survey from 2004 to 2014 (C: North East; D: North West; E: Yorkshire & Humber; F: East Midlands; G: West Midlands; H: Eastern; I&J: London & South East; K: South West). (B) Five ‘Defra regions’ (originally MAFF [Ministry of Agriculture, Fisheries & Food] regions) used in the pesticide usage survey from 1994 to 2002 (1: Northern; 2: Midlands & Western; 3: Eastern; 4: South East; 5: South West). (PDF) [file pone.0223093.s002.pdf]

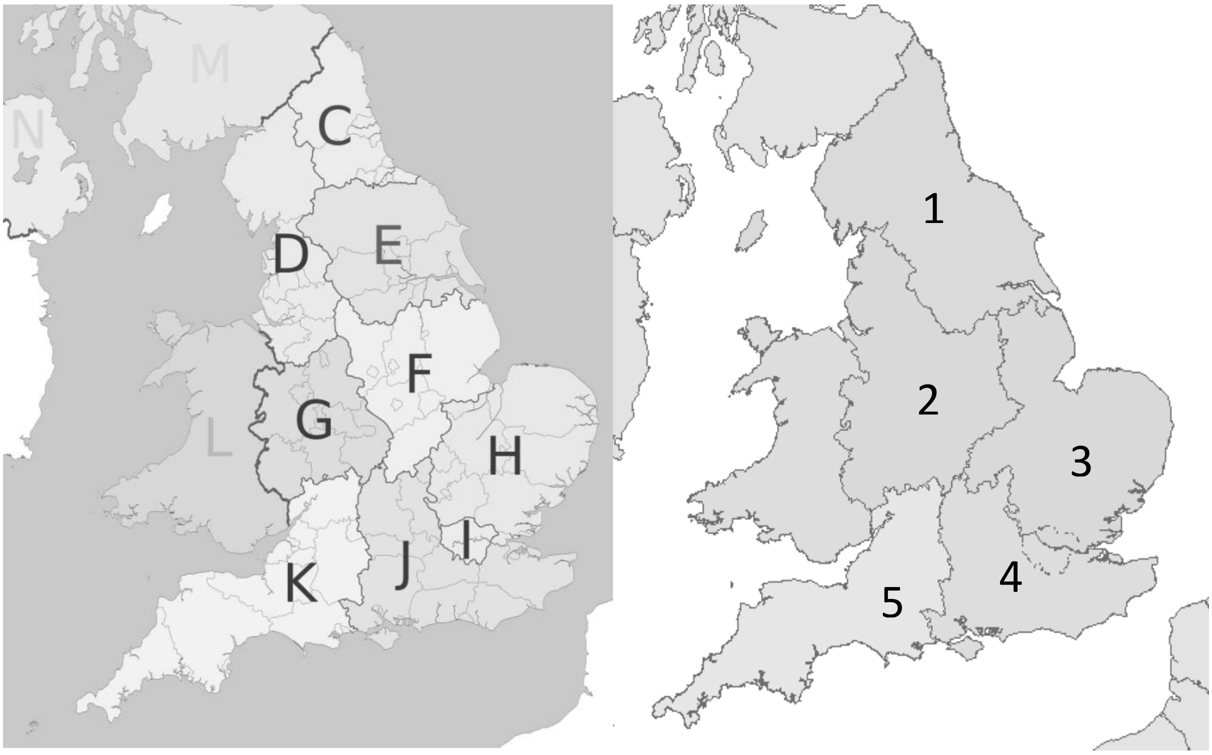

S2 Fig. Pesticide usage survey regions.
(A) Eight 'NUTS regions' (NUTS level 1) used in the pesticide usage survey from 2004 to 2014 (C: North East; D: North West; E: Yorkshire & Humber; F: East Midlands; G: West Midlands; H: Eastern; I&J: London & South East; K: South West). **(B)** Five 'Defra regions' (originally MAFF [Ministry of Agriculture, Fisheries & Food] regions) used in the pesticide usage survey from 1994 to 2002 (1: Northern; 2: Midlands & Western; 3: Eastern; 4: South East; 5: South West).
