# Supplementary material for: Using long-term datasets to assess the impacts of dietary exposure to neonicotinoids on farmland bird populations in England
Source: PLoS One. 2019 Oct 1;14(10):e0223093. doi: 10.1371/journal.pone.0223093 (PMC6772096; doi:10.1371/journal.pone.0223093)
Supplement: S1 Supplementary Note — (PDF) [file pone.0223093.s003.pdf]

S1 Supplementary Note. Interpolation method and validation

To create a dataset to input into the model, cropping data for each of the five main crop types (wheat *Triticum sp.*, winter barley *Hordeum sp*., sugar beet *Beta sp.*, linseed *Linum sp.*, oilseed rape *Brassica sp.*) were required on an annual basis at a 5x5 km resolution for the whole of England. The AgCensus (AgC) data (provided by EDiNA) has these data at a 5x5 km resolution for England, however, it does not provide data for all years between 1994-2014 (**Table A**). For years where these data were missing, two different interpolation approaches were used to estimate cropping areas within each 5x5 km grid. PUS data assigned the cropping category 'set aside' were excluded from the analysis due to the ambiguity of the crop type they were applied to.

Approach 1: June Survey of Agriculture

Regional data from the June Survey of Agriculture (JSA) were used to estimate yearly increases and decreases per crop type from the last available annual AgC data set. Missing years were then interpolated by multiplying the last available AgC annual data ('the baseline') by the increase or decrease in cropping area from the baseline to the JSA data for that missing year. Only the years that had available JSA data and no AgC data were interpolated using this method. Due to an anomaly in the algorithm applied by EDiNA, the regional 2010 AgC cropping data values were consistently below the regional JSA figures (EDiNA, *pers. comm.*). Therefore all AgC data for 2010 were also adjusted (using the same interpolation method), so that it was in accordance with JSA regional cropping data.

Oilseed rape was the only exception to the interpolation protocol in that all AgC years from 2000 (inclusive) onwards were adjusted so that regional AgC data for those years matched JSA regional data. This was due to mis-matches between AgC data and JSA data, which was most likely caused by changeable groupings of oilseed rape categories (e.g., some years all oilseed rape was summed, whereas other years it was divided into two categories based on whether it was winter or spring sown).

Approach 2: linear interpolation

Cropping areas for individual grid squares for any remaining years that had neither AgC nor JSA data were estimated using a linear interpolation using the '`na.approx`' function in the 'zoo' package (2).

Limitations

For each crop type, two 'dummy' years (1995 from 1994 baseline, 2010 from 2004 baseline) that had available AgC data were interpolated using JSA data ('Approach 1'). Interpolated cropping areas were compared to actual AgC cropping areas to assess the effectiveness and accuracy of interpolation methods. We found that grid squares with higher cropping areas produced poorer interpolations, as did missing years that were further away from the last available baseline year. For example, r^2 values for wheat and oilseed rape when comparing actual AgC data to interpolated AgC data for 1995 (created from a 1994 baseline) were 0.93 and 0.85 respectively, whereas the r^2 values for wheat and oilseed rape when comparing actual AgC data to interpolated AgC data for 2010 (created from a 2004 baseline) were 0.77 and 0.64. To account for this, the most recent baseline available was always used and the regional totals for interpolated data for missing years for all crop types were subsequently checked to ensure that they tallied with regional JSA totals for that year. If any grid squares exceeded a total cropping area of 25 km^2, all crops were evenly scaled down so that the total cropping area was capped at 25 km^2. This only occurred for 42 grid squares (range: 2502 – 3453 ha) in any one year across the whole interpolated dataset.

Table A. Data source and interpolation method used for each year of cropping data included in the study (excluding 1998 and 2001).

Crop	Year	AgC	JSA	Baseline	Linear
Wheat & Winter Barley	1994	X			
Wheat & Winter Barley	1995	X			
Wheat & Winter Barley	1996	X			
Wheat & Winter Barley	1997	X			
Wheat & Winter Barley	1998				X
Wheat & Winter Barley	1999		X	1997	
Wheat & Winter Barley	2000	X			
Wheat & Winter Barley	2001		X	2000	
Wheat & Winter Barley	2002		X	2000	
Wheat & Winter Barley	2003	X			
Wheat & Winter Barley	2004	X			
Wheat & Winter Barley	2005		X	2004	
Wheat & Winter Barley	2006		X	2004	
Wheat & Winter Barley	2007		X	2004	
Wheat & Winter Barley	2008		X	2004	
Wheat & Winter Barley	2009		X	2004	
Wheat & Winter Barley	2010	X	X*	2010	
Wheat & Winter Barley	2011		X	2010	
Wheat & Winter Barley	2012		X	2010	
Wheat & Winter Barley	2013		X	2010	
Wheat & Winter Barley	2014		X	2010	
Sugarbeet	1994	X			
Sugarbeet	1995	X			
Sugarbeet	1996	X			
Sugarbeet	1997	X			
Sugarbeet	1998				X
Sugarbeet	1999				X
Sugarbeet	2000	X			
Sugarbeet	2001				X
Sugarbeet	2002				X
Sugarbeet	2003	X			
Sugarbeet	2004	X			
Sugarbeet	2005		X	2004	
Sugarbeet	2006				X
Sugarbeet	2007				X
Sugarbeet	2008				X
Sugarbeet	2009				X
Sugarbeet	2010	X	X*	2010	
Sugarbeet	2011		X	2010	
Sugarbeet	2012		X	2010	
Sugarbeet	2013		X	2010	
Sugarbeet	2014		X	2010	

(*) Indicates which dataset was used where both JSA and AgC data were available.

AgC: agcensus data (5x5 km grid square resolution); JSA: June Survey of Agriculture data (regional resolution); Baseline: AgC data used for JSA interpolation.

Table A (cont.). Data source and interpolation method used for each year of cropping data included in the study (excluding 1998 and 2001).

Crop	Year	AgC	JSA	Baseline	Linear
Oilseed rape	1994	X			
Oilseed rape	1995	X			
Oilseed rape	1996	X			
Oilseed rape	1997	X			
Oilseed rape	1998				X
Oilseed rape	1999		X	1997	
Oilseed rape	2000	X			
Oilseed rape	2001		X	2000	
Oilseed rape	2002		X	2000	
Oilseed rape	2003	X	X*	2003	
Oilseed rape	2004	X	X*	2004	
Oilseed rape	2005		X	2004	
Oilseed rape	2006		X	2004	
Oilseed rape	2007		X	2004	
Oilseed rape	2008		X	2004	
Oilseed rape	2009		X	2004	
Oilseed rape	2010	X	X*	2010	
Oilseed rape	2011		X	2010	
Oilseed rape	2012		X	2010	
Oilseed rape	2013		X	2010	
Oilseed rape	2014		X	2010	
Linseed	1994	X			
Linseed	1995	X			
Linseed	1996	X			
Linseed	1997	X			
Linseed	1998				X
Linseed	1999				X
Linseed	2000	X			
Linseed	2001				X
Linseed	2002				X
Linseed	2003	X			
Linseed	2004	X			
Linseed	2005		X	2004	
Linseed	2006				X
Linseed	2007				X
Linseed	2008				X
Linseed	2009				X
Linseed	2010	X	X*	2010	
Linseed	2011				X
Linseed	2012				X
Linseed	2013				X
Linseed	2014				X

(*) Indicates which dataset was used where both JSA and AgC data were available.

AgC: agcensus data (grid square resolution); JSA: June Survey of Agriculture data (regional resolution); Baseline: AgC data used for JSA interpolation.
