# Supplementary material for: Using long-term datasets to assess the impacts of dietary exposure to neonicotinoids on farmland bird populations in England
Source: PLoS One. 2019 Oct 1;14(10):e0223093. doi: 10.1371/journal.pone.0223093 (PMC6772096; doi:10.1371/journal.pone.0223093)
Supplement: S2 Supplementary Note — (PDF) [file pone.0223093.s004.pdf]

S2 Supplementary Note. Concentrations of neonicotinoid in invertebrates

Although a significant amount of neonicotinoid (NN) is taken up into plants from treated seed, the majority of applied compound is available for transfer out of the seed coating into the surrounding soil. Therefore there is potential for NN to leach into the surrounding substrate and water table when using seed coating as an application method. Using standardised CTD application rates for wheat, calculations were made as to the amount of uptake in soil-dwelling invertebrates, based on earthworms as a model species. The number of seeds sown per unit area (m^2), the ingestion rate of soil for earthworms and type of soil were taken into account. Results from these calculations estimated that the concentration of CTD in earthworms would be <0.0001 ng/g.

Currently, field data for concentrations of NNs measured in 'above-ground' invertebrate samples are largely limited to honeybees (genus: *Apis*), which do not predominate the majority of farmland bird diets (1). One such study reported imidacloprid concentrations in honeybees to be between 0.3 and 11.1 ng/g (2). Data are also available for the concentration of imidacloprid found on multiple species of insect (ground- and canopy-dwelling) as part of the European Food Safety Authority bird and mammal risk assessment for NNs; however these data refer to concentrations of NN measured in insects after imidacloprid was applied via spray treatment, rather than as a seed treatment (3). A over 90% of NN applications in the UK are in the form of seed treatments (4), these data did not inform our study.

Routes of exposure of NNs to birds via invertebrates would be confined to those insects that feed on treated and/or contaminated plants (whether these be wild or crop species), and restricted by the level of residue within each individual plant and the ecology of the insect species (5), which would mediate the level of NN taken up within the invertebrate (e.g., ingesting plant material vs. use as a habitat only). Furthermore, the concentration of NN the bird is subject to would also be dependent on the ratio of exposed:non-exposed invertebrates consumed, the proportion of the diet that

consists of invertebrate species and seasonal/daily changes in foraging habits. Based on the information available with regards to seed treatments and NN concentrations in insect prey items for birds, the ingestion of either above-ground or soil-dwelling invertebrates was considered to be negligible in terms of NN exposure and therefore categorised as a low-residue food item.

References

1. Holland JM, Hutchison MAS, Smith B, Aebischer NJ. A review of invertebrates and seed-bearing plants as food for farmland birds in Europe. Ann Appl Biol. 2006;148(1):49-71.

2. Chauzat MP, Martel AC, Cougoule N, Porta P, Lachaize J, Zeggane S, et al. An assessment of honeybee colony matrices, *Apis mellifera* (*Hymenoptera: Apidae*) to monitor pesticide presence in continental France. Environ Toxicol Chem. 2011;30(1):103-111.

3. European Food Safety Authority. Initial risk assessment provided by the rapporteur Member State Germany for the existing active substance IMIDACLOPRID of the third stage (part A) of the review programme referred to in Article 8(2) of Council Directive 91/414/EEC. Draft Assessment Report (public version). 2006;3(Annex B-9: Ecotoxicology):793-1120.

4. Agriculture and Horticulture Development Board. The neonicotinoid insecticide debate. 2013 Mar 17 [cited March 2017]. Available from: https://horticulture.ahdb.org.uk/news-item/neonicotinoid-insecticide-debate-0.

5. DEFRA. Improved estimation of pesticide residues on arthropods consumed by mammals and birds – validation of semifield data. Department for Environment, Food & Rural Affairs; 2007. Contract No.: PS2323.
