# Supplementary material for: Using long-term datasets to assess the impacts of dietary exposure to neonicotinoids on farmland bird populations in England
Source: PLoS One. 2019 Oct 1;14(10):e0223093. doi: 10.1371/journal.pone.0223093 (PMC6772096; doi:10.1371/journal.pone.0223093)
Supplement: S3 Supplementary Note — (PDF) [file pone.0223093.s005.pdf]

S3 Supplementary Note. Data extraction protocol to inform dietary risk categories

All values in **Table A** were extracted from Tables 1-4 of Holland et al., 2006 (*A review of invertebrates and seed-bearing plants as food for farmland birds in Europe*) (1). Interpolated values for chick diet were calculated by finding the change in total plant material (%) between chicks and adults (breeding or non-breeding, depending on availability), and then estimating the percentage of NN plant material in chick diet based on the change in total plant material from adult to chick. The same approach was used to calculate breeding adult values for skylark.

Table A. Extracted and interpolated dietary values from Holland *et al.,* 2006 (extracted from tables 1-4).

Species included in study	Proportion values extracted for each plant family for each life stage											T1: Total plant material in diet (%)		
	T2: BR Adults				T3: NB Adults				T4: Chicks (%)					
	AM	*CR*	*PO*	*Total*	*AM*	*CR*	*PO*	*Total*	*CR*	*PO*	*Total*	*Br*	*NB*	*N/C*
Chaffinch	0	0	44	44	0	0	25	25			n/a	85	95	
Corn Bunting	0	0	44	44	0	0	75	75		16	16	85		13
Goldfinch	0	0	0	0	0	0	0	0			n/a	95	99	
Greenfinch	0	0	16	16	0	0	11	11	0	21	21	95	99	95
Grey Partridge	0	0	12	12	0	0	28	28	0	21	21	88	100	30
House Sparrow	0	0	37	37	0	0	23	23			*24	6		4
Linnet	0	0	0	0	0	0	0	0	51	20	71	99	99	99
RL Partridge				n/a	11	0	33	44			*29		100	65
Reed Bunting	0	0	0	0	17	0	52	69			*0	39	100	0
Rook			38	38			58	58		34	34	42	78	18
Skylark				*22	36	0	0	36			**2	60	100	^6
Stock Dove	0	29	32	61	0	0	22	22	0	5	5			100
Tree Sparrow	0	0	22	22	14	0	22	36			*15	4	60	5
Turtle Dove	27	41	31	99				n/a	32	38	70	100		100
Woodpigeon	0	18	32	50	0	7	38	45			*47	98	95	97
Yellowhammer			92	92			32	32		4	4	23		35

Grey shading indicates where values are not available.
*Interpolated values calculated from the difference between total plant material (%) in chick and NB *or* Br diet for each species using extracted values for plant families.
**Interpolated using NB value.
^Range given in literature as 0-6.
Plant families data were extracted for: AM: *Amaranthacae* - includes sugarbeet; CR: *Cruciferae* (crops) - includes oilseed rape; PO: *Poaceae* (crops) - includes wheat, barley, oats, rye.
BR: breeding; NB: non-breeding; N/C: nestlings/chicks; T: table; RL: red-legged.

Where data were unavailable in Holland *et al.,* data were extracted from the relevant volumes of Birds of the Western Palearctic (2). For jackdaw (*Corvus monedula*) and starling (*Sturnus vulgaris*) data were extracted as follows - jackdaw (adult): percentage wet weight of cereal grain in 439 stomachs collected

in Spain (all year round) [vol. 8, pg. 126, Table A, Soler *et al.,* 1990] (2); jackdaw (chick): percentage volume of cereals in 357 collar samples collected in Wales [vol. 8, pg. 126, Table B, Richford 1978] (2); starling (chick): absence of crop material in chick diet across multiple studies [vol. 8, pg. 244, Table B] (2)). For kestrel (*Falco tinnunculus*), lapwing (*Vanellus vanellus*), yellow wagtail (*Motacilla flava*) and whitethroat (*Sylvia communis*) the full list of items listed in the 'Food' section for each species were examined. If crop plant material (seed and seedling) did not appear in this list, then a value of zero was given to that species for each life stage.

Data for adult starling were not available from the Birds of the Western Palearctic, so were extracted from Tait *et al.,* 1973 (3) (Appendix II). Percentage values for non-breeding adults were averaged for months outside of April-July, and averaged for months April-July for breeding adults to provide the final values presented in **Table 3** of the main text.

References

1. Holland JM, Hutchison MAS, Smith B, Aebischer NJ. A review of invertebrates and seed-bearing plants as food for farmland birds in Europe. Ann Appl Biol. 2006;148(1):49-71.

2. Cramp S. Handbook of the Birds of Europe the Middle East and North Africa. The Birds of the Western Palearctic, vol. I-IX.: Oxford University Press, Oxford, UK; 1985.

3. Tait MJ. Winter Food and Feeding requirements of the Starling. Bird Study. 1973;20(3):226-236.
