# Supplementary material for: Using long-term datasets to assess the impacts of dietary exposure to neonicotinoids on farmland bird populations in England
Source: PLoS One. 2019 Oct 1;14(10):e0223093. doi: 10.1371/journal.pone.0223093 (PMC6772096; doi:10.1371/journal.pone.0223093)
Supplement: S1 Table — (PDF) [file pone.0223093.s006.pdf]

S1 Table. Summary of species diet (related to high-residue food items), species traits, model input and model output for each of the 22 species included in the analysis.

Species	Latin	Dietary data related to high-residue food items				Species traits			Model input		Model output					
		Adult BR (%)	Adult NB (%)	Chick (%)	Exp. group	Weight (g)	Status^	BBS trend* (%)	BBS sites (N)	Grid square (N)	Model	Estimate	SE	p-value	ODR	RMSE
Chaffinch	*Fringilla coelebs*	44	25	n/a	Med	21	Green	-11	3716	2478	QP	0.000836	0.000130	**<0.001**	0.93	4.58
Corn Bunting	*Miliaria calandra*	44	75	16	High	46.5	Red	-33	**635**	**533**	QP	0.000449	0.000542	0.407	1.25	2.25
Goldfinch	*Carduelis carduelis*	0	0	n/a	Low	15.5	Green	132	3476	2386	QP	**-0.000285**	0.000226	0.207	0.98	3.30
Greenfinch	*Carduelis chloris*	16	11	21	Med	28.5	Green	-51	3355	2327	QP	0.000846	0.000218	**<0.001**	1.04	3.59
Grey Partridge	*Perdix perdix*	12	28	21	Med	400	Red	-58	1387	1130	QP	0.000976	0.000432	**0.024**	0.67	1.11
House Sparrow	*Passer domesticus*	37	23	24	Med	22	Red	-17	2967	2140	QP	**-0.000922**	0.000222	**<0.001**	0.93	7.98
Jackdaw	*Corvus monedula*	n/a	n/a	11	Med	245	Green	68	3408	2333	QP	**-0.000164**	0.000253	0.517	1.24	**10.24**
Kestrel	*Falco tinnunculus*	0	0	0	Low	245	Amber	-20	2952	2095	P	0.000481	0.000293	0.100	0.81	0.60
Lapwing	*Vanellus vanellus*	0	0	0	Low	225	Red	-26	2343	1715	QP	0.000722	0.000396	0.069	**1.68**	6.44
Linnet	*Carduelis cannabina*	0	0	71	High	17.5	Red	-19	2997	2145	QP	0.001252	0.000280	**<0.001**	1.15	4.84
Red-legged Partridge	*Alectoris rufa*	n/a	44	29	Med	475	Green	3	2122	1593	QP	**-0.001437**	0.000252	**<0.001**	0.74	1.75
Reed Bunting	*Emberiza schoeniclus*	0	69	0	High	18.5	Green	44	1641	1287	QP	0.000609	0.000331	0.066	0.84	1.25
Rook	*Corvus frugilegus*	38	58	34	High	490	Green	-13	3209	2242	QP	0.001687	0.000294	**<0.001**	1.14	**27.25**
Skylark	*Alauda arvensis*	22	36	2	Med	39	Red	-23	3347	2293	QP	**-0.000298**	0.000143	**0.038**	0.97	3.46
Starling	*Sturnus vulgaris*	n/a	n/a	0	Med	82.5	Red	-61	3271	2288	QP	0.001210	0.000249	**<0.001**	**1.92**	**20.71**
Stock Dove	*Columbus oenas*	61	22	5	High	310	Amber	22	2654	1969	QP	0.000036	0.000292	0.903	1.47	3.01
Tree Sparrow	*Passer montanus*	22	36	15	Med	22	Red	64	**772**	**666**	QP	0.001692	0.000743	**0.023**	0.83	2.56
Turtle Dove	*Streptopelia turtur*	99	n/a	70	High	155	Red	-94	**775**	**635**	QP	**-0.002093**	0.000534	**<0.001**	0.74	0.95
Whitethroat	*Sylvia communis*	0	0	0	Low	15	Green	25	3035	2157	QP	**-0.000200**	0.000205	0.328	0.85	1.94
Woodpigeon	*Columbus palumbus*	50	45	47	High	515	Green	36	3698	2482	NB	0.000787	0.000160	**<0.001**	1.11	**21.06**
Yellow Wagtail	*Motacilla flava*	0	0	0	Low	20	Red	-42	**851**	**723**	QP	0.000557	0.000456	0.221	0.75	1.42
Yellowhammer	*Emberiza citrinella*	92	32	4	High	27	Red	-28	2676	1918	QP	0.000448	0.000212	**0.035**	0.85	2.34

Numbers in bold indicate those species with significantly fewer data points (BBS sites, Grid Square), species with negative estimates (Estimate), models that are over dispersed (ODR) and species with estimates that have a p-value of < 0.05 (p-value), and species with RMSE >10 (RMSE).

^Status of UK birds as defined by the RSPB according to Birds of Conservation Concern.

*BBS Trend: change in species populations in England between 1995 and 2016 obtained from 'BTO / JNCC / RSPB Breeding Bird Survey Trends 2017 – England' (37).

BR: breeding; Exp.: exposure; NB: non-breeding; BBS: Breeding bird survey; SE: Standard error; ODR: Over dispersion ratio; RMSE: root mean squared error; QP: quasi-Poisson; P: Poisson; n/a: not available.
