# Supplementary material for: Using long-term datasets to assess the impacts of dietary exposure to neonicotinoids on farmland bird populations in England
Source: PLoS One. 2019 Oct 1;14(10):e0223093. doi: 10.1371/journal.pone.0223093 (PMC6772096; doi:10.1371/journal.pone.0223093)
Supplement: S2 Table — A) 'Stepped' interpolation for those years where pesticide surveys did not take place (all odd years). Neonicotinoid usage was assumed to be the same in years where no data were available, as the preceding year (rather than being estimated by a linear interpolation). These data are presented as a means to test the sensitivity of the model to interpolation approaches used. B) Chronic toxicity values used to calculate the toxicity equivalency factor (TEF). Chronic LOAEL values (at the 5% tail of acute sensitivity distribution for avian species) were used (rather than acute LD50 values for bobwhite quail Colinus virginianus) to calculate the TEF for the three compounds included in the study. Calculations were based on information provided in Table 3.2 of Mineau & Palmer (2013). These data are presented as a means to test the sensitivity of the model to differences between acute and chronic TEFs. (PDF) [file pone.0223093.s007.pdf]

S2 Table. **Alternative model outputs for each of the 22 species included in the study. A) 'Stepped' interpolation for those years where pesticide surveys did not take place (all odd years).** Neonicotinoid usage was assumed to be the same in years where no data were available, as the preceding year (rather than being estimated by a linear interpolation). These data are presented as a means to test the sensitivity of the model to interpolation approaches used. **B) Chronic toxicity values used to calculate the toxicity equivalency factor (TEF).** Chronic LOAEL values (at the 5% tail of acute sensitivity distribution for avian species) were used (rather than acute LD50 values for bobwhite quail *Colinus virginianus*) to calculate the TEF for the three compounds included in the study. Calculations were based on information provided in Table 3.2 of Mineau & Palmer (2013). These data are presented as a means to test the sensitivity of the model to differences between acute and chronic TEFs.

**Species**	**Latin**	**Model**	**Model output (A): stepped interpolation**					**Model output (B): chronic TEF**				
			Estimate	*SE*	*p-value*	*ODR*	*RMSE*	*Estimate*	*SE*	*p-value*	*ODR*	*RMSE*
Chaffinch	*Fringilla coelebs*	QP	0.000802	0.000126	**<0.001**	0.94	4.58	0.000841	0.000124	**<0.001**	0.93	4.58
Corn Bunting	*Miliaria calandra*	QP	0.000397	0.000529	0.453	1.25	2.25	0.000483	0.000530	0.362	1.25	2.25
Goldfinch	*Carduelis carduelis*	QP	**-0.000307**	0.00022	0.163	0.98	3.3	**-0.000283**	0.000215	0.189	0.98	3.30
Greenfinch	*Carduelis chloris*	QP	0.000840	0.000212	**<0.001**	1.04	3.59	0.000795	0.000209	**<0.001**	1.04	3.59
Grey Partridge	*Perdix perdix*	QP	0.000907	0.000425	**0.033**	0.67	1.11	0.001015	0.000420	**0.016**	0.67	1.11
House Sparrow	*Passer domesticus*	QP	**-0.000920**	0.000214	**<0.001**	0.93	7.98	**-0.000923**	0.000214	**<0.001**	0.93	7.98
Jackdaw	*Corvus monedula*	QP	**-0.000172**	0.000245	0.481	1.24	10.24	**-0.000188**	0.000240	0.433	1.24	**10.24**
Kestrel	*Falco tinnunculus*	P	0.000470	0.000289	0.104	0.81	0.6	0.000546	0.000282	0.053	0.81	0.60
Lapwing	*Vanellus vanellus*	QP	0.000663	0.000388	0.088	1.68	6.44	0.000605	0.000380	0.111	**1.68**	6.44
Linnet	*Carduelis cannabina*	QP	0.001217	0.000273	**<0.001**	1.15	4.84	0.001409	0.000268	**<0.001**	1.15	4.84
Red-legged Partridge	*Alectoris rufa*	QP	**-0.001422**	0.000243	**<0.001**	0.74	1.75	**-0.001407**	0.000244	**<0.001**	0.74	1.75
Reed Bunting	*Emberiza schoeniclus*	QP	0.001488	0.000335	**<0.001**	0.84	1.25	0.000661	0.000320	**0.039**	0.84	1.25
Rook	*Corvus frugilegus*	QP	0.001615	0.000285	**<0.001**	1.14	27.25	0.001617	0.000280	**<0.001**	1.14	**27.25**
Skylark	*Alauda arvensis*	QP	**-0.000308**	0.000139	**0.027**	0.97	3.46	**-0.000245**	0.000138	0.076	0.97	3.46
Starling	*Sturnus vulgaris*	QP	0.001207	0.000242	**<0.001**	1.9	20.71	0.001177	0.000239	**<0.001**	**1.92**	**20.71**
Stock Dove	*Columbus oenas*	QP	**-0.000045**	0.000283	0.874	1.4	3.01	**-0.000010**	0.000280	0.973	1.47	3.01
Tree Sparrow	*Passer montanus*	QP	0.001687	0.000728	**0.021**	0.83	2.56	0.001649	0.000713	**0.021**	0.83	2.56
Turtle Dove	*Streptopelia turtur*	QP	**-0.002318**	0.000526	**<0.001**	0.74	0.95	**-0.002071**	0.000524	**<0.001**	0.74	0.95
Whitethroat	*Sylvia communis*	QP	**-0.000215**	0.000198	0.276	0.85	1.94	**-0.000152**	0.000195	0.437	0.85	1.94
Woodpigeon	*Columbus palumbus*	NB	0.000769	0.000155	**<0.001**	1.11	21.06	0.000735	0.000153	**<0.001**	1.11	**21.06**
Yellow Wagtail	*Motacilla flava*	QP	0.000507	0.000444	0.254	0.75	1.42	0.000623	0.000445	0.162	0.75	1.42
Yellowhammer	*Emberiza citrinella*	QP	0.000390	0.000206	0.058	0.85	2.34	0.000511	0.000204	**0.012**	0.85	2.34

Numbers in bold indicate those species with negative estimates (Estimate), models that are over dispersed (ODR) and species with estimates that have a p-value of < 0.05 (p-value), and species with RMSE >10 (RMSE). LOAEL: lowest-observed-adverse-effect level; SE: Standard error; ODR: Over dispersion ratio; RMSE: root mean squared error; QP: quasi-Poisson; P: Poisson; n/a: not available.
