# Supplementary material for: Using long-term datasets to assess the impacts of dietary exposure to neonicotinoids on farmland bird populations in England
Source: PLoS One. 2019 Oct 1;14(10):e0223093. doi: 10.1371/journal.pone.0223093 (PMC6772096; doi:10.1371/journal.pone.0223093)
Supplement: S3 Table — (PDF) [file pone.0223093.s008.pdf]

S3 Table. Estimated total application of NN (weight and TEF-adjusted weight) in each region for the entire study period (1994-2014).

**Region**	**NN compound applied (kg)**			**NN compound applied (TEF-adjusted kg)**			**Total NN applied**		**Mean NN applied per grid square**
	IMI	*CTD*	*THX*	*IMI*	*CTD*	*THX*	*(kg)*	*(TEF-kg)*	*(TEF-kg)*
East	995972	544676	171654	995972	43574	17165	1712302	1056712	4.40
East Midlands	426371	234438	26709	426371	18755	2671	687518	447797	2.87
London & South East	279468	259986	0	279468	20799	0	539454	300267	0.85
North East	25567	76540	0	25567	6123	0	102107	31690	0.54
North West	11149	51217	0	11149	4097	0	62366	15246	0.09
South West	105335	221653	0	105335	17732	0	326987	123067	0.48
West Midlands	209659	210885	0	209659	16871	0	420544	226530	1.75
Yorkshire & Humber	125050	142407	3288	125050	11393	329	270745	136772	1.14

CTD: clothianidin; IMI: imidacloprid; NN: neonicotinoid; THX: thiamethoxam; TEF: toxicity equivalency factor.
